# Engineering microbial consortia for biotechnology: An annotated selection of World Wide Web sites relevant to the topics in microbial biotechnology

**DOI:** 10.1111/1751-7915.12273

**Published:** 2015-02-25

**Authors:** Lawrence P Wackett

**Affiliations:** * McKnight Professor, Department of Biochemistry Molecular Biology and Biophysics, BioTechnology Institute, University of MinnesotaSt. Paul, MN, 55108, USA

## Engineering microbial consortia

http://www.cell.com/trends/biotechnology/abstract/S0167-7799(08)00171-6

This important review article helped start a trend of synthetic biologists focusing on using mixed cultures to engineer interspecies metabolism and regulatory networks.

## Synthetic fungal-bacterial consortia

http://www.pnas.org/content/110/36/14592.short

In nature, fungi and bacteria team up to transform cellulose into end products. This particular report describes cellulose conversion to isobutanol.

## Consortia mediated biofuels production

http://www.biotechnologyforbiofuels.com/content/6/1/59

This report describes a yeast-bacterial consortium that transforms cellulose to ethanol.

## Towards synthetic consortia for bioprocessing

http://www.researchgate.net/publication/221885494_Towards_synthetic_microbial_consortia_for_bioprocessing

This Research Gate page describes a review article on broad principles for engineering microbial consortia for practical purposes.

## Engineering and analyzing multi-cellular systems

http://www.springer.com/chemistry/biotechnology/book/978-1-4939-0553-9

This e-book provides an engineering view of using mixed prokaryotic species for carrying out industrially relevant processes.

## Design of microbial consortia for industrial biotechnology

http://yoric.mit.edu/design-microbial-consortia-industrial-biotechnology

This report from a conference proceedings describes a chemical engineering approach to designing multi-species systems for robust industrial biotechnology.

## Unstructured modeling of a synthetic microbial consortia

http://www.ifac-papersonline.net/Detailed/64335.html

This report describes an association between *Trichoderma* and *Saccharomyces* species for degrading cellulose and converting the monomers to ethanol.

## Microbial consortia engineering for cellular factories

http://journals.sfu.ca/rncsb/index.php/csbj/article/view/csbj.201210017/188

This report contains some nice examples of naturally-occurring consortia and synthetic, or engineered, consortia that carry out useful biotransformations.

## Engineering microbial consortia for biomining

http://journal.frontiersin.org/Journal/10.3389/fmicb.2012.00203/full

This Frontiers report largely focuses on mixed microbial populations for handling metals and metal remediation.

## Synthetic ecology to optimize biogas production

http://www.eastscotbiodtp.ac.uk/using-synthetic-ecology-optimise-methanogenic-consortia-anaerobic-digestion

Biogas production is a logical choice for investigating anaerobic microbial consortia that produce methane since methanogens are often highly metabolically interdependent with other microorganisms in natural settings.

## Co-culture technologies

http://rsif.royalsocietypublishing.org/content/11/96/20140065

This is a comprehensive review of co-cultures for biotechnology, has many illustrative figures, and over one hundred references.

## Consortia of cyanobacteria and bacteria

http://www.academia.edu/7450923/Consortia_of_cyanobacteria_microalgae_and_bacteria_Biotechnological_potential

The benefits of cyanobacteria and heterotroph consortia are obvious, with the ability to harvest energy from light and having oxygen transfer from phototroph to heterotroph.

## Stabilizing mixed populations

http://2014.igem.org/Team:Edinburgh/logic/

This IGEM project page describes the design of a control system for working with mixed populations of bacteria.

